# Constraints to exclusive breastfeeding practice among breastfeeding mothers in Southwest Nigeria: implications for scaling up

**DOI:** 10.1186/1746-4358-7-5

**Published:** 2012-04-23

**Authors:** Ojo M Agunbiade, Opeyemi V Ogunleye

**Affiliations:** 1 Department of Sociology and Anthropology, Obafemi Awolowo University, Ile-Ife, Nigeria

**Keywords:** Breastfeeding practices, Exclusive breastfeeding, Social support, Yoruba people

## Abstract

**Background:**

The practice of exclusive breastfeeding is still low despite the associated benefits. Improving the uptake and appropriating the benefits will require an understanding of breastfeeding as an embodied experience within a social context. This study investigates breastfeeding practices and experiences of nursing mothers and the roles of grandmothers, as well as the work-related constraints affecting nurses in providing quality support for breastfeeding mothers in Southwest Nigeria.

**Methods:**

Using a concurrent mixed method approach, a structured questionnaire was administered to 200 breastfeeding mothers. In-depth interviews were also held with breastfeeding mothers (11), nurses (10) and a focus group discussion session with grandmothers.

**Results:**

Breastfeeding was perceived as essential to baby's health. It strengthens the physical and spiritual bond between mothers and their children. Exclusive breastfeeding was considered essential but demanding. Only a small proportion (19%) of the nursing mothers practiced exclusive breastfeeding. The survey showed the major constraints to exclusive breastfeeding to be: the perception that babies continued to be hungry after breastfeeding (29%); maternal health problems (26%); fear of babies becoming addicted to breast milk (26%); pressure from mother-in-law (25%); pains in the breast (25%); and the need to return to work (24%). In addition, the qualitative findings showed that significant others played dual roles with consequences on breastfeeding practices. The desire to practice exclusive breastfeeding was often compromised shortly after child delivery. Poor feeding, inadequate support from husband and conflicting positions from the significant others were dominant constraints. The nurses decried the effects of their workload on providing quality supports for nursing mothers.

**Conclusion:**

Breastfeeding mothers are faced with multiple challenges as they strive to practice exclusive breastfeeding. Thus, scaling up of exclusive breastfeeding among mothers requires concerted efforts at the macro, meso and micro levels of the Nigerian society.

## Background

Breastfeeding benefits for newborns and infants are well-documented [1]. Breastfeeding provides infants with superior nutritional content that is capable of improving infant immunity and possible reduction in future health care spending [2,3]. At the Innocenti Declaration in 1990, the WHO/UNICEF called for policies that would cultivate a breastfeeding culture that encourages women to breastfeed their children exclusively for the first 6 months of life and then up to 2 years of age and beyond [2,4]. However, a recent estimate by the WHO showed that worldwide only 35% of children between birth and their 5th month are breastfed exclusively [5]. Based on the WHO Global data on Infant and Young Child Feeding in Nigeria, 22.3% of children were exclusively breastfed for less than 4 months, while 17.2% were exclusively breastfed for less than 6 months, in the year 2003. According to the Nigerian Demographic and Health Survey (NDHS), in 2008 17% of children were exclusively breastfed for less than 4 months, while 13% were exclusively breastfed for less than 6 months. The median exclusive breastfeeding period in Southwest Nigeria by months in the year 2003 was 7 months. In the year 2008, it was 6 months. Within the same period, early initiation of breastfeeding among women in the region was 12.7% in 2003, but increased to 35.5% in the year 2008 [6]. All these figures are far below the 90% level recommended by the WHO [7]. Child mortality remains high in low and middle-income countries [8]. Nigeria has the highest under-five rural mortality rate of 242.7 per 1,000 among selected sub-Saharan Africa countries [9].

Successful breastfeeding is crucial to the curbing of infant malnutrition and achieving the millennium development goals four [reducing child mortality] and five [improving maternal health] [5]. Based on available evidence, achievements of both goals are still far from the desired progress [10,11]. Breastfeeding practices, including initiation and duration, are influenced by multiple interwoven factors which include health, psychosocial, cultural, political, and economic factors [12,13]. Among these factors, decisions regarding initiation and duration of breastfeeding in low-income countries are influenced by education, employment, place of delivery, family pressure, and cultural values [5,14-17]. In Nigeria, while breastfeeding initiation is on the increase, the duration, and practice of exclusive breastfeeding among women who had their delivery in a health facility, and outside such facility, has remained low [15]. The early introduction of complementary feeding, based on erroneous assumptions, affects breastfeeding initiation and sustainability [5]. Among the Yoruba people, a common belief around infant feeding is that exclusive breastfeeding is beneficial to both infants and mothers, but complementary feeding is essential for babies to adapt to other meals with ease [18-20]. Besides normative expectations, personal experiences and networks of support have influence on the forms and quality of breastfeeding practices. Largely, these factors exert pressure on breastfeeding mothers thereby making their experience pleasurable or painful within time and space [12,21,22].

As an embodied experience, breastfeeding practices and experiences are context bound and culture dependent [23]. Despite the available body of knowledge on breastfeeding practices in Nigeria, studies interrogating the agency of breastfeeding mothers as lived within their socio-cultural context are limited. Earlier, Spencer argued that exploring mothers' breastfeeding experiences as defined within a social context could reveal the inherent complexities mitigating the successfully promotion of sustainable breastfeeding practices [23]. Similarly, Oweis, Tayem, and Froelicher decried the inadequacy of studies investigating breastfeeding mothers' perspectives on breastfeeding barriers and promoting healthy breastfeeding practices [24]. Hence, this study adopts a mixed method design in exploring nursing mothers' breastfeeding practices, beliefs, experiences, and constraints to exclusive breastfeeding. The experiences and views of breastfeeding mothers were complemented with that of grandmothers and nurses. Grandmothers are an important source of support for new mothers. Based on their roles, their infant feeding experience and knowledge can influence mothers' decisions to initiate and continue breastfeeding [25].

## Methods

### Research design and study setting

A complementary mixed method design that consists of a structured questionnaire, in-depth interview, and focus group discussion was adopted. The study population consisted of breastfeeding mothers, nurses and grandmothers. The inclusion criteria for the breastfeeding mothers include ethnic affiliation (Yoruba extraction), having a living child whose age was between three months and one year; and currently seeking postnatal care at the urban comprehensive health centre. Nurses attached to the pediatrics unit of the health facility were invited for the interviews. Grandmothers that are of the Yoruba extraction and had provided or were currently providing informal nursing care within the last one and half years were recruited. In the quantitative phase of this study, the structured questionnaire was the dominant method; while face-to-face in-depth interviews and focus group discussion were adopted in the qualitative phase to enhance, as well as clarify, the quantitative results generated in the survey [26]. This decision was influenced by the nature of the research objectives and the position that mixed methods provide researchers the opportunity to understand social reality from different research paradigms [27].

The survey among breastfeeding mothers and the in-depth interviews with nurses were carried out between February and April 2009 at an Urban Comprehensive Primary Health Centre (PHC) in Ile-Ife, Southwest Nigeria. The Urban Comprehensive Health Centre is under the auspices of the Obafemi Awolowo University Teaching Hospital, Ile-Ife in Osun state. Some of the nurses at the health facility had been trained in exclusive breastfeeding through the Baby-Friendly Hospital Initiative (BFHI) sponsored by the United Nations Children's Fund (UNICEF). The focus group discussion was also conducted in April 2009 at a preferred location suggested by the grandmothers in Ile-Ife. The study location is a semi-urban settlement in Southwest Nigeria. The town had an estimated population of 355,818 during the 2006 census [28]. Despite the availability of modern health care facilities, socio-cultural and economic factors have sustained the relevance of traditional birth attendants in the provision of primary care services in many Nigerian communities [29-31].

### Sampling procedure

Respondents in the quantitative strand were recruited using convenience-sampling technique. All the breastfeeding mothers presented at the Urban Comprehensive Health Centre for scheduled clinic visits from December 2008 to March 2009 were invited to participate in the study. Of this population, 254 breastfeeding mothers who met the study inclusion criteria agreed to participate. Four nurses at the health facility assisted with recruitment of potential respondents. We focused on the breastfeeding experiences and practices of mothers seeking care at a modern health facility, although there are variations in breastfeeding initiation and practices in the wider population of breastfeeding mothers. Of the 254 nursing mothers who met the inclusion criteria, only 224 volunteers responded to the interviewer-administered questionnaire. Twenty four breastfeeding mothers declined participation because they felt they had already spent too much time at the health facility or because they needed to attend to domestic tasks. Hence, the analysis was based on 200 valid responses, representing a response rate of 79 percent.

In the qualitative strand, a purposive sampling approach was used in recruiting 10 nurses in the pediatrics unit of the health facility. Based on the findings from the survey, 17 breastfeeding mothers who had practiced exclusive breastfeeding for a period of six months were invited for in-depth interview, out of which 11 of them honoured the invitation. With the help of a female gatekeeper (individuals who have control over subjects of interest), 10 grandmothers that met the study inclusion criteria were recruited for the focus group discussion.

### Instruments

Three research instruments consisting of questionnaire, in-depth interview and focus group discussion guide were adopted to explore the breastfeeding beliefs, practices and challenges of nursing mothers. A two-page modified version of the questionnaire used in a study on breastfeeding practices of Jordanian women by Oweis et al. was adopted [24]. Three pediatricians and a community health expert assessed the content validity of the questionnaire. Prior to the main study, a pre-test of the instrument was undertaken among 25 nursing mothers recruited from Oke-otubu PHC, Akarabata PHC, and Itasin PHC. All the three PHCs are situated in Modakeke-Ife, a neighbouring community to Ile-Ife. This was to check whether the questionnaire was understandable and pragmatic. The questionnaire included three sections. The first comprised questions that elicit respondents' biographical information i.e. age, level of education, place of child birth, occupation, marital status, sex of the baby, whether pregnancy was planned or not. The second section comprised questions on breastfeeding knowledge, intention, and factors encouraging or discouraging breastfeeding mothers from the practice of exclusive breastfeeding. In this section, 12 questions were targeted at women's breastfeeding behaviour after birth with specific questions on knowledge about breastfeeding initiation, duration, infant nutrition, and challenges of breastfeeding.

In the qualitative phase, additional insights into some of the responses elicited from the survey were sought through face-to-face structured interviews and a focus group discussion session. Generally, qualitative methods provide in-depth information such as participants' perceptions and experiences. Despite the emphasis on depth, qualitative studies are often limited in scope [32]. The face-to-face interview with breastfeeding mothers was focused on breastfeeding experiences and network of supports, while the interviews with the nurses investigated their job experiences and available supports that could facilitate breastfeeding initiation and duration among breastfeeding mothers attending the hospital. Lastly, the focus group discussion with grandmothers was on grandmothers' knowledge, experiences, and dispositions towards exclusive breastfeeding. Two experts in linguistics at the Obafemi Awolowo University, Ile-Ife Nigeria were employed. One translated the three-research instrument from English to Yoruba language, and the other translated it back into English.

### Methods of data analysis

The quantitative data was analysed using the Statistical Package for Social Scientists (SPSS) version 13. Percentages were used to describe the demographic and socioeconomic situation of the women and the breastfeeding beliefs and practices of the women. The findings are presented as both discussions and tables. The in-depth interviews with nurses were conducted in English at the health facility, whilst the interviews with nursing mothers and the focus group discussion with grandmothers were conducted in Yoruba language at preferred locations. All the in-depth interviews and focus group discussion were recorded through audiotape. The first author moderated all the sessions, while the second author acted as the note taker. The interviews lasted for an average of 47 minutes, while the focus group discussion lasted for one hour ten minutes. All the interviews were transcribed verbatim. The interviews that were conducted in Yoruba were transcribed and translated into English by an expert in both languages. Both texts were later given to another expert in linguistics to ensure proper and actual translation. Subsequently, the audio taped interviews and the field notes were used to verify the transcribed texts, ensuring that they were correctly transcribed to preserve the meaning of the participants' words. All the transcribed and translated texts were coded. Coding took place in two stages: open and axial coding. These codes were re-coded, based on the major themes of the study, to form categories. The contents in these categories were used to clarify and enhance the quantitative results [26,33].

### Ethical consideration

Institutional approval was received from the Obafemi Awolowo University Research and Ethics Committee. At all levels, participants were briefed on the study objectives and their consent was received either by filling the informed consent forms or by giving verbal approval before administering any of the research protocols. In addition, all the participants were informed of their right to withdraw their participation in the study at any stage [34].

## Results

### Respondents' profiles

More than one third of the nursing mothers in the survey were between 25 and 29 years of age. The average age at marriage among this category of mothers was 22.5 years with a standard deviation of 3.1 years. Forty two percent of the respondents indicated that they had at least one relative living with them. More than two thirds gave birth at a public or private hospital (86%). A summary of the survey respondents' profiles can be seen in Table 1.

**Table 1:** Selected characteristics of the survey respondents (n = 200)

Variable	N	%
**Age(years):**		

20-24	38	19

25-29	89	45

30-34	55	28

35-39	18	9

**Age at marriage (years) (mean): **22.5, S.D: 3.1		

**Women living with extended families**	84	42

**Religion**		

Christianity	132	66

Islam	68	34

**Level of education**		

No education	10	5

Primary	28	14

Secondary	130	65

Tertiary	32	16

**Occupation**		

Artisan	43	22

Employed in Private Sector	15	8

Employed in Public Sector	25	13

Self employed	30	15

Student	19	10

Trading	39	20

**Place of delivery**		

Public hospital	105	53

Private hospital	68	34

Traditional birth attendant/mission homes	27	14

**Parity**		

Primiparous	76	38

Multiparous	124	62

**Planned pregnancy**	147	74

**Gender of baby**		

Male	93	47

Female	107	54

**Age of baby(months)**		

Less than 3	81	41

3 to 6	55	28

7 to 10	46	23

11 to 12	18	9

### Interviewees and Focus Group Discussant profiles

Among the breastfeeding mothers who were interviewed, the mean age was 24.8 years. The mean age of the 10 nurses was 39.8 years; while that of the 10 grandmothers was 64.6 years. At the time of the focus group discussion, three of the grandmothers were currently providing informal care for their grandchildren whose ages were less than 10 months. Only two among all the grandmothers were able to describe exclusive breastfeeding correctly, even though all of them had breastfeeding knowledge and experiences to varying degrees.

### Breastfeeding practices

A high level of awareness of breastfeeding (94%) and the intention of the mothers to breastfeed their babies up to a year was recorded (73%). Paradoxically, of the mothers whose infants were less than or up to 6 months (n = 136), only 19 percent of them practiced exclusive breastfeeding. Breastfeeding initiation immediately after birth was 45 percent, 29 percent did so within the first two hours after birth. On the length of breastfeeding per feed, more than one third (35%) of the mothers did not take count. Similarly, many of the breastfeeding mothers in the in-depth interview stated that they do not take notice of the duration of breastfeeding for each feed, especially at night. Child breastfeeding at night was common (87%) among the survey respondents (Table 2). Nine among the breastfeeding mothers interviewed described breastfeeding while sleeping as an exercise that requires caution. Most of them admitted that they sleep in the same bed as their infants so that they can breastfeed their babies at any time. In the same vein, all the grandmothers argued:

**Table 2:** Breastfeeding practices

Variable	N	%
**When did you start to feed your baby?**		

Immediately after birth	90	45

Within the first 2 h after birth	58	29

After more than 2 h of birth	35	18

On the second day after birth	11	6

Cannot remember	6	3

**Received information about breastfeeding**		

Yes	187	92

**Perceived adequacy of information**		

Yes	163	82

**How often do you breastfeed your baby daily?**		

< 6 to 8 times	19	10

6 to 8 times	19	10

> 8 times	129	65

As often as the baby wants	33	17

**Length of time for each breastfeeding**		

Less than half an hour	72	36

Half an hour	24	12

More than half an hour	33	17

I don't count	71	36

Breastfeeding when asleep	174	87

**Exclusive breastfeeding**	26	19

**Give baby supplement other than milk**		

Rarely	8	8

Sometimes	53	51

Often	35	34

Most of the time	8	8

**Period intended to breastfeed baby (months)**		

≥ 6	27	12

7 to 12	146	73

13 to 18	14	7

19 to 24	13	7

*From our experiences, after retiring from the farm or market, breastfeeding at night while asleep could be much easier than while awake and it is still common among mothers to date (FGD with grandmothers)*.

Furthermore, the grandmothers described breastfeeding while sleeping at night as a way of keeping babies asleep with little interruption. This position was substantiated in the narratives of six breastfeeding mothers: *'if the child refuses to sleep at night, it is largely the responsibility of mothers to pacify the baby. This often disrupts our sleep,... an average husband will command you to keep his baby asleep with little or no help'*.

### Factors influencing preference for breastfeeding

With the high level of breastfeeding awareness, it was important to understand the factors that helped the respondents' in arriving at the decision to breastfeed. More than two thirds (99%) of the responses indicated breastfeeding as a normative expectation of being a mother. This may not indicate a lack of understanding of the nutritional benefits of breast milk, as 66 percent of the respondents felt breastfeeding helped babies to grow in a normal pattern. A number of the respondents (43%) also recognized their mother-in-law as an essential figure in deciding on the need for breastfeeding (Table 3). In the FGD, four of the grandmothers recounted how their personal involvement in breastfeeding had helped their daughter-in-law to initiate and continue breastfeeding, although not exclusively, but continued breastfeeding up to a year. However, other grandmothers in the group did not express this same view, as three among them argued against the self-sufficiency of breast milk, especially in the first six months of birth.

**Table 3:** Reasons for choosing breastfeeding*

Variable	N	%
Social norm as a mother	119	99

Helping baby to grow in a normal pattern	132	66

Providing baby with natural immunity	111	56

Is a form of child spacing	68	34

Easy and comfortable	19	10

Returned body to normal	3	2

*Women could give more than one reason

Babies cannot feed on breast milk alone. Newborn infants will require additional supplements such as herbal concoction to guard against infections (Grandmother, aged 68)

Largely, the grandmothers described breastfeeding as an essential way of investing in an infants' health, strengthening the physical and spiritual bond between mothers and their children. Similarly, this form of bond associated with breastfeeding was dominant in the interviews with the breastfeeding mothers. They argued that all women must strive to breastfeed in order to strengthen this bond, especially for the nearest future. While exclusive breastfeeding was not emphasized among the grandmothers, women who breastfeed their children for more than a year were labeled as exemplary models of a good mother. An exemplary mother is *'wura ti kose fii owo raa' *(priceless gold) and to achieve this social status, nursing mothers should sacrifice for their children, especially through breastfeeding.

#### Breastfeeding experiences

Five among the breastfeeding mothers described their breastfeeding experiences as challenging and stressful, especially with male children. However, three other mothers argued that a child's sex might not be the sole determinant in most cases. To this latter group, breastfeeding experiences could be both stressful and pleasurable. A consensus among the breastfeeding mothers was that *'breastfeeding is a test of endurance for a mother and it is a way of showing love to one's child'*. Similarly, many of the grandmothers also described breastfeeding as a necessity. To one of the grandmothers:

*Only women breast can produce milk and for this reason, a mother must breastfeed her infant. Once a woman is pregnant, she must be aware of the fact that she must be awake to breastfeed her child even at night (Grandmother, aged 66)*.

One of the nursing mothers described her experience as:

'*It has not been stressful for me to breastfeed especially at night because it is a necessity that I must do (Breastfeeding Mother, aged 27)*.

A further probe on what helped the breastfeeding mothers to choose breastfeeding showed that encouragement from their own mothers was predominant (84%). Social pressure (67%) and personal determination/experience (65%) attracted similar proportions. Encouragements from husbands and nurses/midwives attracted 51 and 46 percent respectively (Table 4). In the in-depth interviews with nurses, a number of the nurses described their involvement in regular health education on breastfeeding during antenatal and postnatal visits to the health facility. This form of education was described as an essential responsibility that should be undertaken at the household level.

**Table 4:** What helped women choose breastfeeding*

Variable	N	%
Encouragement from mother	31	84

Social pressure on mothers	18	67

My personal determination/experience	24	65

Husband encouraged me	19	51

Nurse and midwife	17	46

Encouragement from mother-law	16	43

Media	13	35

My neighbours encouraged me	11	30

Members of my religious affiliation encouraged me	10	27

*Women could give more than one reason

### Constraints and breastfeeding challenges

With the acknowledgement that breastfeeding is beneficial, a commensurate practice was expected. However, less than 20 percent the mothers in the survey practice exclusive breastfeeding. As indicated in Table 5, multiple factors were implicated as constraints. The qualitative findings also revealed that health-related problems, refusal of breast milk by some children, inadequate feeding, and lactation problems were common constraints to exclusive breastfeeding. A recurrent position in the interviews with breastfeeding mothers and nurses was that the discontinuation of exclusive breastfeeding might be against the desires of some mothers. As described by one of the breastfeeding mothers:

**Table 5:** Reasons for discontinuation of exclusive breastfeeding (n = 167)*

Variable	N	%
Baby continued to be hungry after feeding	49	29

Maternal health problem	45	27

Fear of infant becoming addicted to breast milk	43	26

Due to pains in my breast	41	25

My mother-law pressured me to wean the baby	41	25

I was not making enough breast milk to satisfy my child	40	24

I returned to work/business	28	24

Lack of husband's support	46	23

Breastfeeding was too tiring	37	22

My neighbours pressured me to wean the baby	37	22

Baby refused breast milk	36	22

I was losing weight	33	20

I feel dizzy at times during breastfeeding	30	18

Due to pregnancy	22	13

My baby was not gaining enough weight	19	11

I was not feeding well	19	11

*Women could give more than one reason

*'Some mothers find it very difficult to breastfeed for 6 months. This is because they complain that their breast aches as their babies had to suck hard for the milk to flow steadily. Nevertheless, a breastfeeding mother with the needed support and consumes well-nourished balanced meal, will experience constant secretion of milk. But when such support is lacking and the secretion of milk fluctuates with complaints of breast pain, infant supplement becomes an alternative' (Breastfeeding mother, aged 32)*.

While painful experiences could be a determinant, one of the grandmothers argued otherwise:

*'Some breastfeeding mothers do not breastfeed because they believe it could make their breast flabby and unappealing especially to their husbands. A number of men have high preference for well-shaped breast. Hence, they discontinue breastfeeding at short intervals to discourage their husbands from looking outside *[i.e. engage in extra-marital affairs] (Grandmother, aged 61).

In contrast to the argument depicted in the extract, a number of the participants agreed that the fear of infants becoming addicted to the breast milk, lack of quality support from the husband, and pressure from some grandmothers on their daughter-in-laws could encourage a discontinuation of exclusive breastfeeding. Three among the grandmothers argued that introducing semi-solids or water alongside breast milk would enable a child to grow faster and allow their mothers to resume economic activities without a prolonged break.

However, seven among the nurses recounted how they had encouraged close associates and mothers who visit the Health Centre to practice exclusive breastfeeding. The nurses also confirmed that at the health facility, mothers are often educated on the need to see breastfeeding as an investment that would yield bountiful dividends. Even though this is the usual practice at the health facility, all the nurses expressed dissatisfaction with the rate of exclusive breastfeeding among mothers in the locality. As an approach, a number of the nurses expressed the need for effective interventions at the hospital that could prompt breastfeeding beyond mere early initiation to sustainable exclusive breastfeeding. However, the nurses expressed concerns over their increasing workload pressure and inadequate staffing as factors within the hospital that limited their ability to provide additional support for nursing mothers.

## Discussion

Evidence from this study showed an average level of breastfeeding initiation immediately after childbirth (45%), and within the first two hours after birth (29%). The intention to breastfeed for up to 12 months was high among the respondents (73%). With the focus on mothers currently seeking postnatal care at a modern health facility that is baby friendly, there are likely chances that their awareness and dispositions towards exclusive breastfeeding could differ from the larger population of mothers. For instance, in Davies-Adetugbo's study on awareness and relevance of colostrum among nursing mothers in a rural Yoruba community in Nigeria, colostrum was perceived as milk that had stayed in the breast during the 9 months of pregnancy and thus become stale [35]. While the relevance of colostrum had increased among the breastfeeding mothers in this study, traditional child feeding practices such as feeding infants with herbal concoction are still common among Yoruba people. This may be hinged on some cultural beliefs associated with child birth and rearing practices among the Yoruba people in southwest Nigeria [29,36].

Breastfeeding culture is well enshrined in the various ethnic groups in Nigeria [14], but the low practice of exclusive breastfeeding persists. The Nigerian Demographic Health Survey in Nigeria, 2008 showed that only 13% of children below six months are exclusively breastfed while 87% of Nigerian infants below six months receive complementary liquids or foods. Only 19% (136) of the breastfeeding mothers whose infants were below or up to six months of age in this study practiced exclusive breastfeeding. There are variations in the exclusive breastfeeding rate found in this study compared with others [19,37]. The variations in the prevalence rates may be due to the sample size and design adopted. However, the low practice of exclusive breastfeeding remains a concern, especially with the high level of awareness as revealed in the initiation rate and perceived benefits associated with breastfeeding.

In the Yoruba belief system, the breast is a symbolic part of the woman's body constructed as a vital source of nutrients and a medium of physical and spiritual bond sustenance between the mother and the child. From both the quantitative and qualitative findings, breastfeeding mothers are faced with personal and social constraints in practicing exclusive breastfeeding. Specific constraints identified include maternal health, breast and nipple problems, perceived milk insufficiency, and pressure from significant others. The qualitative findings also revealed that the good mother would struggle to appropriate the physical and spiritual bonds and benefits associated with exclusive breastfeeding. The mismatch between breastfeeding intention and the practice of exclusive breastfeeding indicates the existence of conflict revolving around intentions, normative expectations, and social pressures to practice exclusive breastfeeding among the breastfeeding mothers. These findings support the findings of Otoo, Lartey and Perez-Escamilla on perceived incentives and barriers to exclusive breastfeeding among peri-urban Ghanaian Women [17]. Otoo et al. reported that there was widespread belief that exclusive breastfeeding was easier when breast milk began to flow soon after delivery [17]. The main obstacles to exclusive breastfeeding identified were maternal employment, breast and nipple problems, perceived milk insufficiency, and pressure from family. With the available evidence portraying Nigeria as a challenging place for mothers [38], such conflicts could create additional psychosocial problems for younger mothers in particular, and older mothers who may lack access to adequate quality support networks. In addition, the practice of breastfeeding at night while both mother and child are asleep could create a level of discomfort for the mother and probable risk for the child as admitted in the findings.

In the Yoruba culture, significant others (grandmothers, mothers-in-law, and relations) are actively involved in the production of child health including the sustenance of breastfeeding culture [36]. In this study, grandmothers and mother-in-laws played dual roles in the forms and prevailing breastfeeding practices reported among the respondents. For instance, some grandmothers felt that the early introduction of complementary feeding and herbal concoction would be better than breast milk only. From the participants' experiences and cultural expectations, grandmothers who did not practice exclusive breastfeeding are likely to exert pressure on younger mothers to discontinue exclusive breastfeeding, especially with the occurrence of lactation problems or pressing health challenges affecting either the mother or the child. This finding supports Grassley and Eschiti's position that grandmothers' own infant feeding experience and knowledge can influence mothers' decisions to initiate and continue breastfeeding or not [25]. In Uchendu, Ikefuna and Emodi's study among breastfeeding mothers at the University of Nigeria Teaching hospital, 52% of women who had never practiced exclusive breastfeeding reported family opposition, especially grandmothers, and personal decision-making as major constraints [39]. In spite of the structural constraints, breastfeeding experiences differ among the mothers. Those who practiced exclusive breastfeeding among them perceived the subjective norm of an exemplary mother, the joy attached to childbirth, personal resolutions, spouse, and mother-in-law's support as modifiers and insulators from social and psychological constraints in some respect. The family structure, in the form of extended families, was also considered as supportive in promoting the practice of breastfeeding, especially at the level of early initiation.

As revealed in this study, and similar to findings by Oweis, Tayem and Froelicher [24] and Otoo, Lartey, and Pérez-Escamilla [17], breastfeeding could be tiring, stressful or pleasurable to some mothers. Breastfeeding was described as stressful, painful, or pleasurable based on personal and prevailing circumstances around breastfeeding mothers. Some of the grandmothers and breastfeeding mothers described exclusive breastfeeding as an investment in a child's life. While a mother may intend to practice exclusive breastfeeding for the first six months and continue with breastfeeding for up to year, personal and socio-cultural factors could act as constraints. From the findings in this study, a number of the breastfeeding mothers were confronted with erroneous beliefs and child rearing practices that supported the introduction of herbs and solid food alongside breastfeeding. This is besides the normative responsibilities of motherhood and situational limitations such as poverty, high cost of living and the need to provide support for their household needs.

Similar to the above, and somewhat pressing, is the inadequate feeding among nursing mothers and its implications on the practice of exclusive breastfeeding. This latter constraint requires holistic measures such as empowering mothers economically. More so, the larger involvement of mothers in the provision of economic support of the household is also an indication of pressure on mothers and a possible source of disempowering women in the informal sector of the economy from practicing exclusive breastfeeding. There is an indication that a number of the breastfeeding mothers are undergoing strain as they struggle to engage in economic activities to support their husbands. At the moment, the high poverty level and costs of living in Nigeria expose a lot of households to malnutrition [11]. Hence, we would suggest further investigation on motherhood and breastfeeding experiences of women, especially those with low-socioeconomic backgrounds. Findings from such studies may provide insight into the psychosocial consequences of conforming to cultural notions of motherhood and constraints associated with exclusive breastfeeding.

While counseling and proper education on desirable breastfeeding practices could be adopted to achieve a change in attitudes, perceptions, knowledge and practice of exclusive breastfeeding, the inadequate quality support from health care providers, as illustrated by the experiences of the nurses, could be a challenge. However, this is not peculiar to the provision of educational support on breastfeeding. Inadequate supply of health professionals and increasing health challenges is the bane of modern health delivery in developing nations. The migration of health care professionals and the poor working conditions prevailing in many sub-Saharan African countries has worsened the situation [40].

Hence, designing effective and prompt intervention initiatives that could promote the provision of quality support for nursing mothers' would require concrete efforts from all stakeholders, not just from the hospitals or healthcare system. Such efforts will go a long way in creating a sustainable exclusive breastfeeding culture and bridging the existing gaps in achieving millennium development goals four and five in Nigeria.

One of the limitations of this study is its focus on breastfeeding mothers at a modern health facility, which may have influenced the levels of breastfeeding initiation and practices. Another limitation is the sample design and the sample size selected. While a mixed method design was adopted to strengthen the findings, generalization of the findings is further limited with the existing cultural variations among the various ethnic groups in Nigeria.

## Conclusion

Based on the findings, breastfeeding mothers are faced with multiple challenges as they strive to practice exclusive breastfeeding. Thus, scaling up of exclusive breastfeeding among mothers requires concerted efforts at the macro, meso, and micro levels of Nigerian society. Reversing the existing brain drain in the health sector will require substantial improvements in working conditions, and empowering of healthcare providers to provide improved care. In addition, there is an urgent need of policies that will aim at providing acceptable food supplements that could aid the supply of breast milk among postpartum mothers, especially those with low socio-economic status. Similarly, policies aimed at improving exclusive breastfeeding uptake should also incorporate significant others (grandmothers, mothers-in-laws, and husbands) in the process of encouraging breastfeeding mothers. There is an indication that significant others play active roles in encouraging or discouraging exclusive breastfeeding practices among the study population.

## Competing interests

The authors declare that they have no competing interests.

## Authors' contributions

OMA designed the study, developed the research instruments, managed the qualitative data collection, performed the quantitative and qualitative analysis, drafted and revised the manuscript. OVO managed the quantitative data collection and participated in the interpretation of the data. Both authors read and approved the final manuscript.
